# Bromide of Ethyl in Dental Practice

**Published:** 1891-09

**Authors:** 


### Bromide of Ethyl in Dental Practice.

Probably every one is pleased to learn that our experts are still busy experimenting with anæsthetics. The results given at the last meeting of the Odontological Society were not such as will, we imagine, encourage the adoption of Nunneley's anæsthetic among English dentists. Dr. Silk, who introduced the interesting young stranger, was not able to state a very strong case for him, his appendix showed that in a rather large number of cases more or less untoward symptoms followed its use. However, it must be remembered that some of these were hardly test cases, inasmuch as Dr. Silk had adopted a better method in the latter ones. The surgeons who spoke of their experience in operating upon patients who were under the bromide did not find it satisfactory, as there seemed some doubt when the patient was "under," and the after sickness, as with chloroform and ether, struck them as placing the anæsthetic behind our old familiar friend "the gas." It is curious how nitrous oxide holds its own, at one time cocaine, at another oxygen and other gases crop up, and now bromide of ethyl, but like Tennyson's brook—"gas" 'goes on forever.' The late Mr. Clover thought ethidene dichloride was to oust gas and every thing else, but his vaticination has fallen to the ground. The anæthetists who spoke on Dr. Silk's paper, although several of them had not tried bromide of ethyl in dental practice spoke strongly against its use, and if as was urged by Dr. Dudley Buxton, the action of this anæsthetic is physiologically that of chloroform, its employment in dentistry must be open to the objections usually advanced against that substance.—*Editorial in Dental Record.*

Salt in Milk for Children.—The addition of sodium chloride prevents the solid coagulation of milk by either rennet or gastric juice. The cow's milk ought never to be given without table salt, and the latter ought to be added to a woman's milk when it behaves like cow's milk in regard to solid curdling and consequent indigestibility. Habitual constipation of children is influenced beneficially, since not only is the food made more digestible, but the alimentary secretions, both serous and glandular, are made more effective by its presence.—*Arch. of Ped.*

Sweating of the Hands.—A lotion composed of four ounces of cologne water and a half an ounce of tincture of belladonnæ is highly recommended as a cure for the disagreeable sweating of the hands and feet, from which many persons suffer. The affected extremities should be rubbed two or three times a day with the lotion.

To Remove Iodoform Odor from Hands.—For the removal of the iodoform odor from the hands and utensils, Bienert recommends washing once or twice with linseed oil and water. The odor is said to disappear with surprising quickness..—*Pharm. Centralhalle.—Druggists' Bulletin.*

